# CompMoby: Comparative MobyDick for detection of cis-regulatory motifs

**DOI:** 10.1186/1471-2105-9-455

**Published:** 2008-10-27

**Authors:** Christina Chaivorapol, Collin Melton, Grace Wei, Ru-Fang Yeh, Miguel Ramalho-Santos, Robert Blelloch, Hao Li

**Affiliations:** 1 Department of Biochemistry and Biophysics, California Institute for Quantitative Biomedical Research, Graduate Program in Biological and Medical Informatics; University of California, San Francisco, San Francisco, CA 94143-2540, USA; 2 Department of Urology, University of California, San Francisco, San Francisco, CA 94143, USA; 3 Institute for Regeneration Medicine, University of California, San Francisco, San Francisco, CA 94143, USA; 4 Diabetes Center, University of California, San Francisco, San Francisco, CA 94143, USA; 5 Department of Epidemiology and Biostatistics, University of California, San Francisco, San Francisco, CA 94107, USA; 6 Departments of Obstetrics/Gynecology and Pathology, University of California, San Francisco, CA 94143, USA

## Abstract

**Background:**

The regulation of gene expression is complex and occurs at many levels, including transcriptional and post-transcriptional, in metazoans. Transcriptional regulation is mainly determined by sequence elements within the promoter regions of genes while sequence elements within the 3' untranslated regions of mRNAs play important roles in post-transcriptional regulation such as mRNA stability and translation efficiency. Identifying cis-regulatory elements, or motifs, in multicellular eukaryotes is more difficult compared to unicellular eukaryotes due to the larger intergenic sequence space and the increased complexity in regulation. Experimental techniques for discovering functional elements are often time consuming and not easily applied on a genome level. Consequently, computational methods are advantageous for genome-wide cis-regulatory motif detection. To decrease the search space in metazoans, many algorithms use cross-species alignment, although studies have demonstrated that a large portion of the binding sites for the same trans-acting factor do not reside in alignable regions. Therefore, a computational algorithm should account for both conserved and nonconserved cis-regulatory elements in metazoans.

**Results:**

We present CompMoby (Comparative MobyDick), software developed to identify cis-regulatory binding sites at both the transcriptional and post-transcriptional levels in metazoans without prior knowledge of the trans-acting factors. The CompMoby algorithm was previously shown to identify cis-regulatory binding sites in upstream regions of genes co-regulated in embryonic stem cells. In this paper, we extend the software to identify putative cis-regulatory motifs in 3' UTR sequences and verify our results using experimentally validated data sets in mouse and human. We also detail the implementation of CompMoby into a user-friendly tool that includes a web interface to a streamlined analysis. Our software allows detection of motifs in the following three categories: one, those that are alignable and conserved; two, those that are conserved but not alignable; three, those that are species specific. One of the output files from CompMoby gives the user the option to decide what category of cis-regulatory element to experimentally pursue based on their biological problem. Using experimentally validated biological datasets, we demonstrate that CompMoby is successful in detecting cis-regulatory target sites of known and novel trans-acting factors at the transcriptional and post-transcriptional levels.

**Conclusion:**

CompMoby is a powerful software tool for systematic *de novo *discovery of evolutionarily conserved and nonconserved cis-regulatory sequences involved in transcriptional or post-transcriptional regulation in metazoans. This software is freely available to users at .

## Background

The increasing number of sequenced genomes of multicellular eukaryotes, including human, along with high-throughput methods such as whole genome microarray expression data, allows for systematic characterization of the cis-regulatory elements that control gene expression. Regulation of gene expression occurs at multiple levels in metazoans, including transcriptional and post-transcriptional. Transcriptional regulation involves binding of transcription factors (TFs) to short cis-regulatory elements, or transcription factor binding sites (TFBSs), that are generally 5–15 basepairs (bp) long. TFs bind to specific TFBSs on DNA, which leads to activation or repression of gene transcription [1] into mRNA. mRNA stability and translation efficiency may be further regulated at the post-transcriptional level. One of the most well studied forms of post-transcriptional regulation involves binding of microRNAs (miRNAs) to cis-regulatory target sites residing in the 3' untranslated regions (UTRs) of mRNA and results in translational repression [2,3]. The above mechanisms of regulation of gene expression are the most well studied, but other forms of regulation can also be deciphered at the DNA level, such as targets of RNA binding proteins (RBPs) [4-7].

Existing experimental methods to identify cis-regulatory elements, or motifs, are time-consuming and cannot easily be scaled up to analyze a large number of genes [1]. Some other techniques for large scale analysis of sequences, such as chromatin immunoprecipitation-on-chip (ChIP-chip), require prior knowledge of the trans-acting factor (see [8] for review of experimental techniques). In contrast, certain types of computational algorithms can be used to discover *de novo *motifs in the noncoding regions (NCRs) on a genome-wide scale without prior knowledge of the trans-acting factor and the labor costs of experimental techniques. NCRs are defined as any DNA sequence residing outside the translational start and end site of all known genes of a genome. In general, motifs are more difficult to detect in metazoans because the overall genomes are much larger than yeast, including the NCR (~200 times larger in human compared to yeast, where ~95% of the human genome is noncoding). Furthermore, regulatory elements can reside far upstream, downstream, in introns [1], or UTR [3] regions of the genes they regulate. The larger sequence search space leads to an increase in background noise and more difficulty in detecting true regulatory elements. There are existing computational algorithms that identify TFBSs (see [8] and [9] for reviews) and miRNA targets (see [10] for review). To identify TFBSs, most algorithms start out with a set of co-regulated genes that are functionally related, which may be obtained using microarray data, to search for enriched motifs [1]. Many of these algorithms were developed and tested in yeast, where the intergenic regions are much shorter and the motifs less degenerate than in metazoans. Many miRNA target prediction programs in metazoans begin with known miRNAs (computationally and/or experimentally identified) and use specific base-pairing rules between the miRNA and its targets to predict putative binding sites [3,10].

Algorithms for detecting motifs in metazoans tend to leverage evolutionary conservation to reduce the background noise [1,10]. There may be a disadvantage for algorithms that solely rely on alignments as input because studies have shown that the conservation of cis-regulatory sites tend to be low for the same trans-acting factor across different species [3,6,11]. Multiple studies have shown that TFBSs often do not fall within conserved regions [11-13]. Odom et al. demonstrated that approximately two-thirds of the binding sites of orthologous genes between human and mouse for the same transcription factor occurred in sequences that did not align. Due to the lack of verified miRNA binding sites, studies have not been as extensive in studying turnover of target sites across species. However, recent studies indicate there is approximately 50% conservation of miRNA targets between human and mouse, and that 30–50% of nonconserved sites might be functional in human when the miRNA and mRNA are expressed in the same tissue [3]. Alignment algorithms also have their own set of technical problems such as misalignments due to large insertions and/or deletions [3,14], which may affect defining conserved sites as nonalignable. Therefore, computational algorithms that incorporate both sequence conservation and species specific information for detecting cis-regulatory motifs are advantageous.

We previously introduced the CompMoby (Comparative MobyDick) algorithm for the study of transcriptional regulation in metazoans, which was successfully applied to embryonic stem (ES) cells [15]. We have further developed the CompMoby software by developing a friendly user web interface that streamlines the analysis pipeline by formatting the user input and filtering the output with suggested default thresholds. The website also allows the user to download the necessary software for extraction of aligned NCRs that are not easily obtainable from a public database (discussed in the implementation section), includes sample input files for both 5' upstream and 3' downstream sequence analysis, and documentation that explains the results in the output files. The algorithm has also been extended to accommodate analysis of 3' UTRs for post-transcriptional regulatory studies. The utility of this software for 5' upstream and 3' downstream sequence analysis will be demonstrated in the results and discussion section.

The CompMoby software integrates species specific and evolutionary conservation information as input into the MobyDick algorithm [16,17] and formats the output files to systematically identify over-represented putative TFBSs in upstream sequences, or putative miRNA and RBP targets in 3' UTR sequences. Our tool requires no prior knowledge of the trans-acting factor and allows *de novo *motif discovery, which may lead to identification of a new TF, miRNA, or RBP. Currently, we restrict analysis of miRNA and RBP target sites to the 3' UTR sequences since studies have shown that most known metazoan miRNA targets reside in the 3' UTR [2,3].

CompMoby has the advantage of being comprehensive and flexible because the software does not only rely on alignments, and cis-regulatory motifs that are alignable do not have to be 100% conserved. CompMoby can also capture degenerate cis-regulatory sites in its clustering step, where similar motifs that are not necessarily exact matches are grouped together. This flexibility is a useful feature because cis-regulatory sites for both TFs and miRNAs are often degenerate in animals [1,3]. Our algorithm also allows the user to identify multiple putative cis-regulatory motifs of different lengths in one run by capturing both strong and weak motifs without having to iteratively mask the strongest motif in the input sequences [9] or rerunning the algorithm for varying motif widths. The ability to capture multiple motifs of varying width and enrichment is a useful feature because there may be multiple TFBSs per upstream region of a gene [1,18] or multiple miRNAs per mRNA [2,19] that are often involved in combinatorial regulation of gene expression in metazoans. Most existing algorithms use either specific species information or multiple alignments in any one run and output the results separately. In comparison, the CompMoby software automatically combines all the MobyDick dictionaries derived from aligned and non-aligned sequence sets into clusters of motifs and calculates a p-value of over-representation for each cluster.

The CompMoby software provides the user with a tool to identify *de novo *cis-regulatory elements functioning at the transcriptional as well as the post-transcriptional level in metazoans. The nature of the algorithm allows the biologist to systematically identify the exact positions of putative cis-regulatory elements that are conserved and/or species specific on a genome-wide scale for experimental follow-up, which may provide insight into further understanding the complex regulatory networks of metazoans.

## Methods

### Algorithm overview

**Figure 1:**
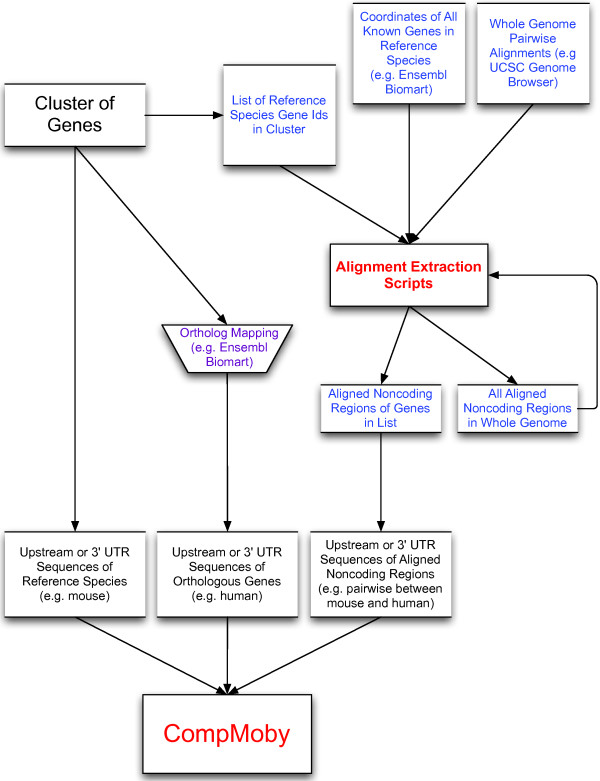
**Flow chart of input data to the CompMoby software**. All three sets of input sequences are derived from a set of genes. External data sources are shown in blue or purple text. The alignment extraction scripts can be downloaded from . The input sequence set containing aligned noncoding regions is produced from the alignment extraction scripts.

**Figure 2:**
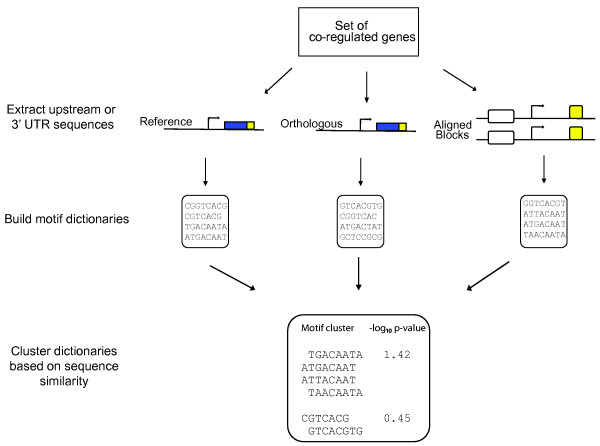
**An overall summary of the CompMoby software data analysis and algorithm design**. Reference refers to upstream or 3' UTR sequences from the same species of the set of co-regulated genes. Orthologous refers to sequences from genes orthologous to the reference species. Aligned blocks are the upstream or 3' UTR conserved sequences derived from a genome-wide pair-wise alignment (obtainable from the UCSC genome browser) between the species used in the reference and orthologous sequences.

**Figure 3:**
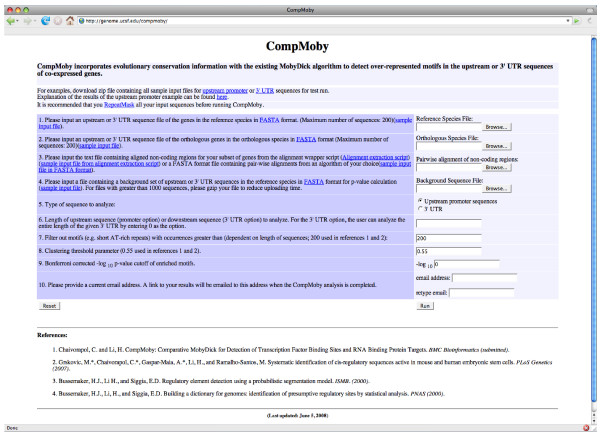
The web-based user interface for the CompMoby software.

**Figure 4:**
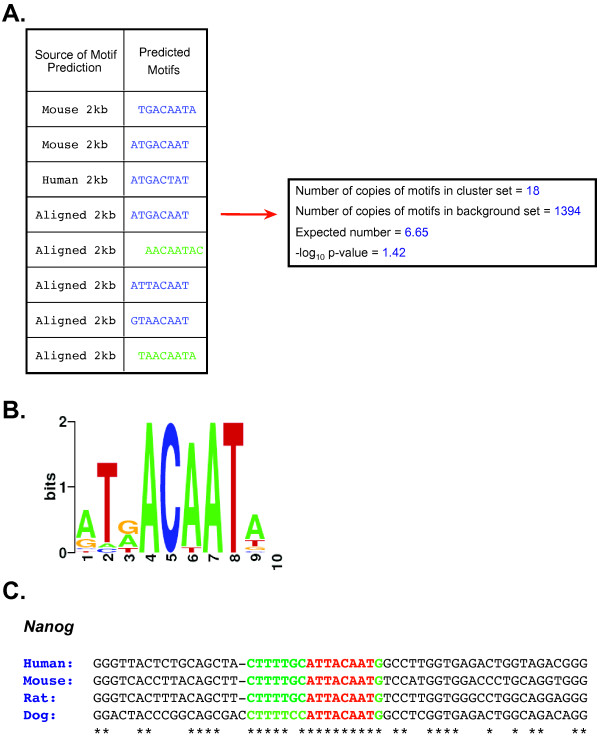
**CompMoby identification of the Oct4 and Sox2 binding site upstream of *Nanog *in mouse embryonic stem cells**. (A) The table shows the sequence source of the motifs in the motif cluster. CompMoby predicted the reverse complement of the motifs in green. The right box shows the number of occurrences of the motif cluster in the 2kb upstream sequences of the co-regulated mouse pluripotent gene set compared to a background set of ~8500 other mouse genes. The expected number is calculated from the number of occurrences in the background set, assuming a random distribution. The -log_10 _p-value is Bonferroni corrected by the total number of motif clusters. (B) WebLogo (v. 2.8) [47] representation of the motif cluster. (C) Four species alignment [27] of upstream region of *Nanog *containing the Oct4 and Sox2 binding sites. Red represents the motif predicted by CompMoby. Green represents the flanking region extended from the predicted motif based on conservation of the nucleotide position in three out of four species. Asterisk (*) represents a fully conserved position.

CompMoby is a general cis-regulatory motif discovery algorithm that can be used to identify motifs enriched in intergenic [15] and 3' UTR sequences. The flow of input data to CompMoby is shown in Figure 1, overall algorithm architecture in Figure 2, and the user interface in Figure 3. This software has been demonstrated to work effectively in mammalian species [15], where upstream regions of length 2kb were used compared to lengths of 600bp typically used in the promoter analysis of yeast. The CompMoby software integrates all the tools necessary to incorporate evolutionary conservation and species specific sequence information as input into the MobyDick algorithm and filters and processes the output into two files: the first contains all motif clusters with their corresponding -log_10 _p-values; the second contains only motif clusters that pass a user defined Bonferroni corrected -log_10 _p-value cutoff and identifies the sequence source (i.e. reference, ortholog, or aligned blocks, described in further detail in the next section) of the motifs within the cluster (Figure 4A).

### Input sequences

The algorithm is based on input sequences derived from a set of co-expressed genes. The input data consists of three sets of RepeatMasked [20] FASTA formatted sequences with Ensembl gene identifiers  (Figure 1)[21]. The sequences can be the upstream regions from the transcriptional start site (TSS) or the 3' UTR sequences of a group of co-expressed genes (e.g. genes with similar temporal or spatial expression patterns). Upstream sequences can be used to identify TFBSs, and 3' UTR sequences to identify targets of miRNAs or RBPs. One set of sequences is derived from the reference species, or the species from which the group of co-expressed genes is obtained. The second set of sequences is obtained from the orthologous genes in another species and the mapping is user defined. Sources for orthologous gene mapping include the Ensembl and UCSC genome browser  databases. The third set of alignable sequences from the reference species is derived from the genome-wide pair-wise alignment between the species used for the reference and orthologous sequences. The pair-wise alignment data can be downloaded from the UCSC genome browser site [22,23]. Only the aligned, or conserved, blocks in the NCRs of the co-expressed genes are extracted for CompMoby analysis. The aligned blocks are only extracted if they lie within the same length upstream or downstream of the gene as the reference species sequence set. The aligned set of sequences reduced the search space by a third in a pair-wise alignment between mouse and human compared to mouse alone using a group of co-expressed genes specific to mouse pluripotent cells [15]. Alternatively, the user may choose to use a pair-wise alignment algorithm of their choice and may input the third set of alignable sequences in FASTA format.

Perl scripts to extract all NCRs from the UCSC genome-wide pair-wise alignment can be downloaded on the CompMoby website at . We provide a wrapper script that implements all necessary scripts for extraction. This script is computationally intensive because it scans the entire genome for NCRs, but only needs to be run once per build for the reference species of interest and the type of sequence analysis (upstream or 3' UTR). Depending on the user specified options, there are three different types of output files that can be produced from the alignment wrapper script: a file containing all NCRs in the entire genome; a file containing NCRs from only a subset of genes if the previous file has already been created; or both of the above files (Figure 1).

The NCRs are either associated with the nearest downstream gene for TFBS sequence analysis or with the nearest upstream gene for 3' UTR analysis. For subsequent runs on the same build and type of sequence analysis, the user may implement the wrapper script to extract only the NCRs of a specified length from a subset of genes. This file containing the subset of aligned NCRs is used as input into CompMoby (Figures 1 and 2). A tab delimited text file containing all known reference species genes using Ensembl gene identifiers and their chromosomal coordinates is required to locate NCRs. This file can be obtained from the Ensembl Biomart feature [24]. For an example of this file, see . The user should make sure that the builds of the reference and orthologous sequences, as well as the Ensembl file of known genes, are the same as those of the pair-wise alignments.

Given the set of input sequences, the user can choose to analyze a certain length of noncoding region (e.g. 1kb upstream from the TSS or 1kb downstream from the 5' end of the 3' UTR). For the 3' UTR analysis, the user has the option to analyze the entire length of the UTR sequences (Figure 3). This feature is only offered in the 3' UTR option since UTR sequences are generally not as long as upstream regions which can be 100's of kbs long, and it would be computationally intensive to search such a large sequence space. The background sequence set, clustering threshold parameter and p-value cutoff options in Figure 3 will be discussed further on.

### Building dictionaries and filtering

Once the set of sequences is obtained, the software inputs these sequences into the MobyDick algorithm [16,17] to build three dictionaries of putative motifs in parallel. The general idea behind the MobyDick algorithm is that DNA sequences are decomposed into a set, or dictionary, of motifs based on a probabilistic segmentation model. The following MobyDick adjustable parameters are used: *L*, the maximum word length, was set to 8, and *MaxP*, the probability of seeing at least one false positive at each testing step, was set to 0.1. Once the three motif dictionaries are obtained, CompMoby filters out unlikely motifs by the following criteria: one, motif length less than 5; two, number of copies greater than a user defined number to eliminate repetitive elements, such as short AT-rich repeats (default is 200); three, a quality factor (which describes how likely the motif is real instead of being made by shorter pieces by chance) to be greater than 0.2 (for details, see materials and methods section of [25]).

From the set of three filtered dictionaries, all the motifs are grouped together by sequence similarity with the CAST clustering algorithm [26] to obtain a single dictionary of motif clusters (Figure 2). All pairs of motifs in the dictionaries are scored for similarity based on a gapless pair-wise alignment using a simple mutation model described in [25]. The gapless pair-wise alignment does not take into account reverse complements for 3' UTR analysis, unlike TFBS analysis, since miRNAs and RBPs targets are single stranded mRNAs. After scoring all pair-wise alignments based on sequence similarity, the CAST algorithm is used to group motifs into clusters, referred to as motif clusters, with the default threshold parameter set at 0.55 (the lower bound of the normalized score averaged over all pairs in a cluster)(Figure 3). A higher threshold parameter will lead to smaller motif clusters, while a lower threshold parameter will lead to larger clusters of motifs with less sequence similarities within each cluster. This clustering step allows for degeneracy of the cis-regulatory sites within and across species.

### Calculating motif cluster enrichment

-log_10 _p-values are calculated from a Poisson distribution based on the number of occurrences of the motif cluster in the input reference species sequence set compared to an expected number of occurrences derived from a random background set. The background set usually consists of the upstream or 3' UTR sequences of the same length as the input data from the entire genome of the reference species, or all other genes on the microarray from which the co-expressed genes were derived. This background sequence set can be obtained using the Ensembl Biomart feature . The user has the flexibility to upload a sequence file in FASTA format containing a background set of their choice. We recommend using a background set consisting of 5000 sequences or less and uploading a gzipped file to the CompMoby web interface due to the upload time. The -log_10 _p-value of each motif cluster is then Bonferroni corrected by the total number of clusters (for details of the p-value calculation, refer to the Materials and Methods section of [15]).

### Output files

**Figure 5:**
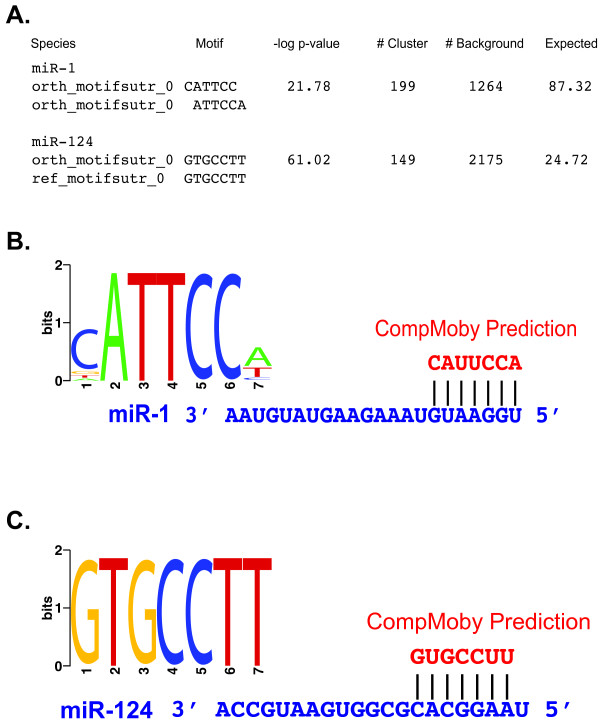
**Identification of mammalian tissue specific miRNA target sites by CompMoby**. (A) Output file format generated by CompMoby given a user defined Bonferroni corrected -log_10 _p-value cutoff of greater than 0. These results represent the top motif cluster from two independent runs of CompMoby on two different sets of co-regulated genes that were significantly down-regulated with miR-1 or miR-124 overexpression in HeLa cells [34]. "Species" column represents the sequence source of the predicted "Motif". "# Cluster" represents the number of occurrences of the motif cluster from the reference species sequence set and "# Background" the number of occurrences in the background sequence set. The "-log_10 _p-value" and "Expected" columns are calculated as described in Figure 3B. (B) WebLogo (v. 2.8) representation of the miR-1 site identified by CompMoby. Also shown is the match between the predicted motif cluster to the miR-1 seed region. U (mRNA) is equivalent to T (DNA). (C) WebLogo (v. 2.8) representation of the miR-124 site identified by CompMoby. Also shown is the match between the predicted motif cluster to the miR-124 seed region.

CompMoby processes the results into two output files: one contains all motif clusters with their corresponding -log_10 _p-values; the second contains only motif clusters that pass a user defined Bonferroni corrected -log_10 _p-value cutoff (default 0)(Figure 3) as well as the input sequence source (i.e. reference, ortholog, or aligned blocks) from which each of the motifs within the motif cluster are derived (Figure 5A). The website address to the output files are emailed to the user when the analysis is completed. Explanation of the output files can be found from the web interface at .

### Implementation and user interface

CompMoby is implemented in Perl and the interface is written in HTML and CGI. The MobyDick algorithm is mainly implemented in C with a Perl wrapper. The CompMoby software and all dependencies were developed and tested on a 2.4 GHz Intel Xeon desktop machine. The CompMoby software can be found at  (Figure 3).

## Results and discussion

### Application to analysis of transcriptional regulation in embryonic stem cells

**Figure 6:**
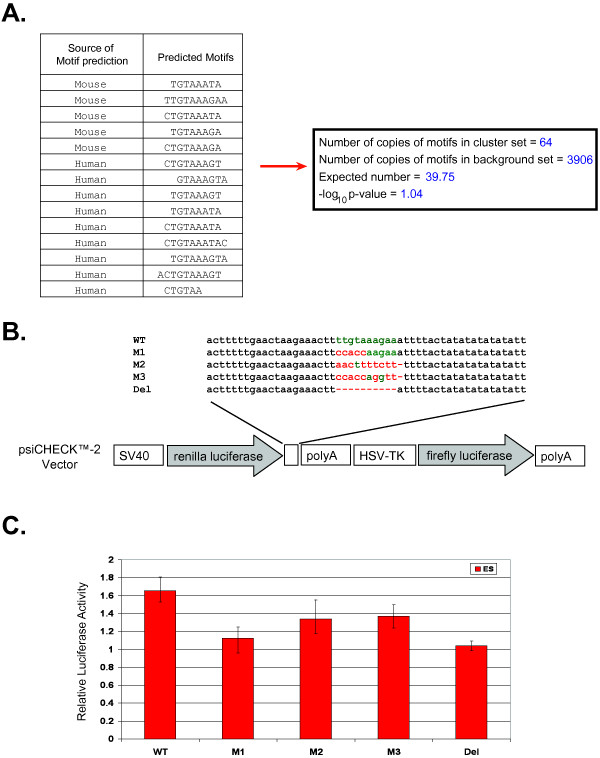
**Experimental validation of regulatory site found in 3' UTR of embryonic stem cell analysis**. (A) Table shows the sequence source of the motifs in the motif cluster. The right box shows the Bonferroni corrected -log_10 _p-value of the motif cluster in the 3' UTR sequences calculated as described in Figure 4A. (B) Schematic representation of reporter constructs used in study. Constructs contain either the predicted motif, which occurs in the 3' UTR of n-*myc *and depicted in green (WT), the mutated motif (M1, M2, M3), or a deletion of the motif (Del). Red nucleotides represent mutations and dashes represent deletions. (C) Regulatory activity of predicted motif in mouse ES cells. Constructs described in (B) were transfected in mouse ES cells. After 14–18 hours, cells were lysed and assayed for luciferase activity. The ratio of Renilla to Firefly luciferase was taken for each experiment. Each experiment was done in triplicate, except M3 which was done in duplicate, and the ratios were averaged. The average values were then normalized to the activity of the empty construct. Bars represent the averages of the normalized values with error bars indicating the range.

In our previous study, we applied CompMoby to a dataset of 41 co-expressed genes (refer to Additional file 1 for accession numbers) that were highly up-regulated in mouse embryonic pluripotent cells compared to differentiated cells to gain insight into the transcriptional regulation of ES cells (for details, see [15]). Part of one motif predicted by CompMoby, ATTACAAT, was extended using a four species alignment [27] into the flanking regions (Figure 4) and experimentally verified by two different labs [28,29] as a binding site for Oct4 and Sox2 upstream of *Nanog*, a key regulator of ES cells (for review on ES cell regulation, see [30]). Experimental validation of 10 out of the 62 predicted motif clusters that passed our Bonferroni corrected cutoff (-log_10 _p-value > 0) from the CompMoby analysis of the pluripotent co-expressed gene set, showed that in mouse ES cells 56% of the tested motifs (different motifs from each of the 10 motif clusters) showed significant luciferase reporter activity compared to a control containing only a basal promoter (t-test, α = 0.05). Interestingly, when the motifs that had significant reporter activity in mouse ES cells compared to differentiated cells were experimentally tested in human ES cells, the luciferase reporter activity was conserved. Four out of the ten motif clusters also demonstrated sequence specific binding by proteins present in ES cells using electrophorectic mobility shift assays (see Figure 6a in reference [15]). It is interesting to note that the reporter activity of a couple of the motifs are comparable to that of the known Oct4 enhancer (positive control), which suggests that they may bind novel TFs with an important role in regulating ES cells. The other bound motif clusters are also novel predictions for which there are no known regulators and may bind TFs with important, yet undiscovered roles in regulating ES cells.

An advantage of using computational tools such as CompMoby over experimental methods is the ability to systematically predict TFBSs and their location upstream of many genes at once, thus reducing the time-consuming experimental dissection of large NCRs. In this particular case of cis-regulatory motif analysis in mammalian species, CompMoby improves upon MobyDick. The Oct4 and Sox2 binding site upstream of *Nanog *would not have been discovered using MobyDick on the mouse sequences alone because the motif is only found in the set of sequences containing aligned blocks between mouse and human (Figure 4A). Another motif cluster shown to be bound by a protein present in ES cells would not have been discovered using only MobyDick since the motif is only found in the aligned blocks sequence set. Other cis-regulatory prediction algorithms such as MEME (version 3.0.10) and Bioprospector (release 1) also failed to identify the Oct4/Sox2 binding site using only the mouse sequences. MEME was independently run eight times with the following adjustable parameters: *nmotifs *= 4, *width *= 6, 8, 10, or *max width *= 14 and *min width *= 5. Bioprospector was independently run 15 times, with five trials per width due to the stochastic nature of the algorithm, with the following adjustable parameters: *width *= 6, 8, or 10 and *background *= same background sequence file used for CompMoby analysis of motif enrichment. We also compared CompMoby to CompareProspector, which uses alignment information from multiple species. CompareProspector (May 2005 web release)[31] discovered a consensus motif that was a 7 out of 8 bp match to part of the experimentally verified Oct4/Sox2 binding site using alignments between mouse and human, while CompMoby found a motif cluster, where one motif within the cluster was an exact match. In order to identify the Oct4/Sox2 binding site, CompareProspector had to be run multiple times with the following adjustable parameters: 1kb or 2kb mouse and human aligned sequence file using LAGAN (version 1.2)[32], *width *= 6, 8, or 10, *conservation threshold *= default values, and *background *= aligned input sequences or 1kb mouse background sequence file used for CompMoby calculation of motif enrichment. Only when CompareProspector was run with *width *= 8 and *background *= aligned input sequences was the Oct4/Sox2 binding site found.

By analyzing both species specific and aligned sequences simultaneously, CompMoby enables a user to decide what type of motif they would like to experimentally test depending on the biological study. This feature is important for regulatory studies in model species, where the user may wish to study conserved as well as divergent regulatory pathways. Our previous study demonstrates the power of CompMoby to identify regulatory motifs that may be functional in the model organism and applicable to human, and to discover novel TFs that may have important roles in regulation by not requiring *a priori *knowledge of known TFs.

### CompMoby identifies the target sites of mammalian tissue-specific miRNAs

miRNAs play an important role in controlling gene expression at the post-transcriptional level and studies have shown that one miRNA may have multiple binding sites on the same target mRNA, which leads to stronger repression of the mRNA [3,33]. In animals, miRNAs match target sites in 3' UTRs of mRNA with imperfect complementary pairing. The seven consecutive nucleotides starting from the first or second bp of the 5' end of a miRNA is the seed region, which is one of the important criteria for determining mRNA target recognition. We reasoned that since the seed region is a motif often occurring in multiple copies in 3' UTR sequences, we could treat miRNA target site prediction similarly to that of TFBSs given a set of co-regulated genes. CompMoby was tested on two human datasets [34] to access the algorithm's ability to correctly identify the miRNA target motif in the 3' UTR sequences of a set of co-expressed genes.

Lim et al. generated the datasets by independently over-expressing miR-1 or miR-124 in human HeLa cells and then profiling the mRNA on whole genome microarrays. Both miR-1 and miR-124 are known for their tissue specificity in mammals, where the former is preferentially expressed in heart and skeletal muscle, while the latter is preferentially expressed in brain. Lim et al. identified 96 genes that were significantly down-regulated (p-value < 0.001) at both 12 and 24 hours with miR-1 over-expression and 174 genes with miR-124 over-expression.

We considered each set of the down-regulated genes to be co-regulated and used the human 3' UTR sequences (build 36) of these genes mapped to Ensembl gene identifiers (release 45) for input data into CompMoby as the reference species (refer to Additional files 2 and 3 for accession numbers). 3' UTR sequences from the orthologous mouse genes (build 36) were used as the orthologous sequence set. The aligned blocks set containing 3' UTR sequences of the co-regulated genes were derived from whole genome pair-wise alignments between human (build 36) and mouse (build 36) downloaded from the UCSC genome browser. For cases where there are multiple transcripts per gene, the longest transcript was used to avoid over counting motif occurrences. The background sequences consisted of all 3' UTRs from the entire human genome minus the set of 3' UTRs from the set of co-expressed genes. CompMoby was instructed to analyze the entire 3' UTR sequences (input field number six, length option, in Figure 3). The motif cluster with the most significant p-value predicted by CompMoby was the target site of miR-1 (Figures 5A, 5B) and miR-124 (Figures 5A, 5C). The input sequence files for the miR-124 over-expression analysis used in the CompMoby analysis can be found at . MobyDick analysis on the human 3' UTR sequences alone would not have been able to identify the target site of miR-1, which is derived from only the mouse sequence set (Figure 5A), where the signal for the target site may be stronger than in human.

### CompMoby identifies a regulatory motif in the 3' UTR of n-*myc *in mouse ES cells

We applied CompMoby to analyze the 3' UTR sequences of 123 co-expressed genes (refer to Additional file 4 for accession numbers) that were 1.5-fold up-regulated in all pair-wise comparisons of different types of mouse pluripotent cells, including ES cells, to various types of differentiated cells (Wei G., Yeh RF., Hebrok M., and Ramalho-Santos M., submitted) to identify putative miRNA and/or RBP target sites. 42 predicted motif clusters passed our Bonferroni corrected cutoff of -log_10 _p-value > 0. To assess the performance of CompMoby on this dataset, we experimentally validated a motif cluster that was derived from the mouse and human sequence sets (Figure 6A) and found in the 3' UTR of n-*myc*. This motif cluster was not found using Bioprospector (release 1) or MEME (version 3.5.7) on the mouse 3' UTR sequences alone. We focused on the n-myc motif because n-*myc *is highly up-regulated in pluripotent cells [35]. Furthermore, there is likely redundancy of the different forms of Myc proteins regulating self-renewal in ES cells [36]. c-*myc *is capable of reprogramming fibroblasts to pluripotent stem cells in combination with other TFs [37-41], but n-*myc *is largely interchangeable with c-*myc *[42] and can substitute for c-*myc *in reprogramming fibroblasts [35]. We transfected mouse ES cells with a Firefly and Renilla luciferase reporter construct containing the motif in the 3' UTR region of the Renilla gene (Figure 6B; see Additional file 5 for experimental materials and methods). The construct contained a single copy of the motif plus a 50bp flanking region. This construct showed a significant increase in luciferase expression when compared to the empty construct (no motif) transfected into mouse ES cells (Figure 6C). To verify that the predicted motif and not the flanking region was driving luciferase expression, we made three different mutants of the motif as well as deleting the motif altogether (Figures 6B and 6C). The increased luciferase expression was significantly reduced or abolished indicating that the predicted motif is responsible for the enhanced activity.

These experimental results suggest that this motif is a functional activator in ES cells. RBPs recognize binding sites on their target mRNAs and may act as an activator or repressor on gene expression by controlling mRNA stability and translation during post-transcriptional regulation [5,6,43,44]. In human neuroblasts, n-*myc *has been demonstrated to be regulated by a RBP that affects the stability of n-*myc *mRNA [7,45,46]. The results from analyzing published datasets and our experimental validation of ES cell data demonstrate that CompMoby can be used to identify cis-regulatory motifs in the 3' UTR sequences of mammalian species given a set of co-regulated genes. CompMoby also allows for discovery of novel miRNAs and other trans-acting factors by assuming no prior knowledge of the trans-acting factor. Many miRNA binding sites are not evolutionarily conserved (~50% binding sites are not conserved between mouse and human) [3] and CompMoby offers the advantage of allowing detection of both conserved and nonconserved binding sites in the 3' UTRs.

### Future directions

With the increasing number of sequenced genomes, future versions of CompMoby will allow for multiple species alignments to be used. Another addition will be the option for users to upload gene identifiers instead of 5' upstream and 3' UTR sequences, which will reduce the upload time and allow for the analysis of a greater number of sequences per species. For the pair-wise alignment sequence set, uploading gene identifiers will eliminate the computationally intensive work of NCR extraction for the user. This additional feature will require storage of sequences from the most commonly used species and recent builds. CompMoby will also be extended to allow for 5' UTR and intronic sequence analysis in future versions.

## Conclusion

CompMoby is a comprehensive and flexible software tool with a user web interface for *de novo *detection of conserved and nonconserved motifs from a set of co-regulated genes. CompMoby is an improvement over MobyDick for cis-regulatory motif detection in mammals by allowing for multiple input sequence files to take advantage of evolutionary conservation information, and filtering the output files into user-friendly formats. Our software is a useful tool that has been demonstrated to successfully identify known and novel motifs in real biological datasets that have been experimentally validated by other studies as well as in our previous study, which may lead to discovery of new TFs, miRNAs, or RBPs. Our results using experimentally validated datasets demonstrate that CompMoby can be used to study regulation at both the transcriptional and post-transcriptional levels in metazoans by systematically identifying regulatory motifs on a genome-wide scale.

## Availability and requirements

Project name: CompMoby

Project home page: .

Operating system(s): Platform independent (web interface: HTML, CGI), UNIX (Perl scripts).

Programming language: Perl, C.

License: Accessible and free for all users.

## Abbreviations

Bp: basepair; ChIP-chip: chromatin immunoprecipitation-on-chip; CompMoby: comparative MobyDick; ES cells: embryonic stem cells; miRNA: microRNA; NCR: noncoding region; RBP: RNA binding protein; TF: transcription factor; TFBS: transcription factor binding site; TSS: transcriptional start site; UTR: untranslated region

## Authors' contributions

CC and HL conceived and designed the project. CC developed and coded CompMoby and web interface, and analyzed and interpreted 5' upstream and 3' UTR sequence results for all datasets. CM and RB designed, performed, and analyzed luciferase reporter experiments, and CM, RB, CC, and HL interpreted luciferase results. RY, GW, and MRS analyzed and provided list of up-regulated pluripotent cell genes for the 3' UTR analysis. CC and HL wrote the manuscript. All authors read and approved the final manuscript.

## Supplementary Material

Additional file 1 **Accession numbers of embryonic stem cells for upstream sequence analysis.** Ensembl accession numbers of genes up-regulated in mouse embryonic stem cell compared with differentiated cells. Click here for file

Additional file 2 **Accession numbers of miR-1 over-expression.** Ensembl accession numbers of genes down-regulated in human HeLa cells with miR-1 over-expression. Click here for file

Additional file 3 **Accession numbers of miR-124 over-expression.** Ensembl accession numbers of genes down-regulated in human HeLa cells with miR-124 over-expression. Click here for file

Additional file 4 **Accession numbers of embryonic stem cells for 3' UTR analysis.** Ensembl accession numbers of genes 1.5-fold up-regulated in all pair-wise comparisons of different types of mouse embryonic pluripotent cells to various types of differentiated cells. Click here for file

Additional file 5 **Materials and methods for luciferase reporter assays.** Materials and methods for ES cell culture and luciferase reporter assays in 3' UTR sequence analysis. Click here for file

## References

[B1] Bulyk ML (2003). Computational prediction of transcription-factor binding site locations. Genome Biol.

[B2] Bartel DP (2004). MicroRNAs: genomics, biogenesis, mechanism, and function. Cell.

[B3] Chen K, Rajewsky N (2007). The evolution of gene regulation by transcription factors and microRNAs. Nat Rev Genet.

[B4] White EK, Moore-Jarrett T, Ruley HE (2001). PUM2, a novel murine puf protein, and its consensus RNA-binding site. Rna.

[B5] Pique M, Lopez JM, Foissac S, Guigo R, Mendez R (2008). A combinatorial code for CPE-mediated translational control. Cell.

[B6] Gerber AP, Luschnig S, Krasnow MA, Brown PO, Herschlag D (2006). Genome-wide identification of mRNAs associated with the translational regulator PUMILIO in Drosophila melanogaster. Proc Natl Acad Sci USA.

[B7] Manohar CF, Short ML, Nguyen A, Nguyen NN, Chagnovich D, Yang Q, Cohn SL (2002). HuD, a neuronal-specific RNA-binding protein, increases the in vivo stability of MYCN RNA. J Biol Chem.

[B8] Elnitski L, Jin VX, Farnham PJ, Jones SJ (2006). Locating mammalian transcription factor binding sites: a survey of computational and experimental techniques. Genome Res.

[B9] D'Haeseleer P (2006). How does DNA sequence motif discovery work?. Nat Biotechnol.

[B10] Sethupathy P, Megraw M, Hatzigeorgiou AG (2006). A guide through present computational approaches for the identification of mammalian microRNA targets. Nat Methods.

[B11] Odom DT, Dowell RD, Jacobsen ES, Gordon W, Danford TW, MacIsaac KD, Rolfe PA, Conboy CM, Gifford DK, Fraenkel E (2007). Tissue-specific transcriptional regulation has diverged significantly between human and mouse. Nat Genet.

[B12] Dermitzakis ET, Clark AG (2002). Evolution of transcription factor binding sites in Mammalian gene regulatory regions: conservation and turnover. Mol Biol Evol.

[B13] Emberly E, Rajewsky N, Siggia ED (2003). Conservation of regulatory elements between two species of Drosophila. BMC Bioinformatics.

[B14] Margulies EH, Cooper GM, Asimenos G, Thomas DJ, Dewey CN, Siepel A, Birney E, Keefe D, Schwartz AS, Hou M (2007). Analyses of deep mammalian sequence alignments and constraint predictions for 1% of the human genome. Genome Res.

[B15] Grskovic M, Chaivorapol C, Gaspar-Maia A, Li H, Ramalho-Santos M (2007). Systematic identification of cis-regulatory sequences active in mouse and human embryonic stem cells. PLoS Genet.

[B16] Bussemaker HJ, Li H, Siggia ED (2000). Regulatory element detection using a probabilistic segmentation model. Proc Int Conf Intell Syst Mol Biol.

[B17] Bussemaker HJ, Li H, Siggia ED (2000). Building a dictionary for genomes: identification of presumptive regulatory sites by statistical analysis. Proc Natl Acad Sci USA.

[B18] GuhaThakurta D (2006). Computational identification of transcriptional regulatory elements in DNA sequence. Nucleic Acids Res.

[B19] Krek A, Grun D, Poy MN, Wolf R, Rosenberg L, Epstein EJ, MacMenamin P, da Piedade I, Gunsalus KC, Stoffel M (2005). Combinatorial microRNA target predictions. Nat Genet.

[B20] Smit A, Hubley R, Green P (2004). RepeatMasker Open-3.0.

[B21] Flicek P, Aken BL, Beal K, Ballester B, Caccamo M, Chen Y, Clarke L, Coates G, Cunningham F, Cutts T (2007). Ensembl 2008. Nucleic Acids Res.

[B22] Karolchik D, Baertsch R, Diekhans M, Furey TS, Hinrichs A, Lu YT, Roskin KM, Schwartz M, Sugnet CW, Thomas DJ (2003). The UCSC Genome Browser Database. Nucleic Acids Res.

[B23] Kent WJ, Baertsch R, Hinrichs A, Miller W, Haussler D (2003). Evolution's cauldron: duplication, deletion, and rearrangement in the mouse and human genomes. Proc Natl Acad Sci USA.

[B24] Hammond MP, Birney E (2004). Genome information resources – developments at Ensembl. Trends Genet.

[B25] Patil CK, Li H, Walter P (2004). Gcn4p and novel upstream activating sequences regulate targets of the unfolded protein response. PLoS Biol.

[B26] Ben-Dor A, Shamir R, Yakhini Z (1999). Clustering gene expression patterns. J Comput Biol.

[B27] Xie X, Lu J, Kulbokas EJ, Golub TR, Mootha V, Lindblad-Toh K, Lander ES, Kellis M (2005). Systematic discovery of regulatory motifs in human promoters and 3' UTRs by comparison of several mammals. Nature.

[B28] Kuroda T, Tada M, Kubota H, Kimura H, Hatano SY, Suemori H, Nakatsuji N, Tada T (2005). Octamer and Sox elements are required for transcriptional cis regulation of Nanog gene expression. Mol Cell Biol.

[B29] Rodda DJ, Chew JL, Lim LH, Loh YH, Wang B, Ng HH, Robson P (2005). Transcriptional regulation of nanog by OCT4 and SOX2. J Biol Chem.

[B30] Boyer LA, Mathur D, Jaenisch R (2006). Molecular control of pluripotency. Curr Opin Genet Dev.

[B31] Liu Y, Liu XS, Wei L, Altman RB, Batzoglou S (2004). Eukaryotic regulatory element conservation analysis and identification using comparative genomics. Genome Res.

[B32] Brudno M, Do CB, Cooper GM, Kim MF, Davydov E, Green ED, Sidow A, Batzoglou S (2003). LAGAN and Multi-LAGAN: efficient tools for large-scale multiple alignment of genomic DNA. Genome Res.

[B33] Grimson A, Farh KK, Johnston WK, Garrett-Engele P, Lim LP, Bartel DP (2007). MicroRNA targeting specificity in mammals: determinants beyond seed pairing. Mol Cell.

[B34] Lim LP, Lau NC, Garrett-Engele P, Grimson A, Schelter JM, Castle J, Bartel DP, Linsley PS, Johnson JM (2005). Microarray analysis shows that some microRNAs downregulate large numbers of target mRNAs. Nature.

[B35] Blelloch R, Venere M, Yen J, Ramalho-Santos M (2007). Generation of induced pluripotent stem cells in the absence of drug selection. Cell Stem Cell.

[B36] Cartwright P, McLean C, Sheppard A, Rivett D, Jones K, Dalton S (2005). LIF/STAT3 controls ES cell self-renewal and pluripotency by a Myc-dependent mechanism. Development.

[B37] Maherali N, Sridharan R, Xie W, Utikal J, Eminli S, Arnold K, Stadtfeld M, Yachechko R, Tchieu J, Jaenisch R (2007). Directly reprogrammed fibroblasts show global epigenetic remodeling and widespread tissue contribution. Cell Stem Cell.

[B38] Okita K, Ichisaka T, Yamanaka S (2007). Generation of germline-competent induced pluripotent stem cells. Nature.

[B39] Takahashi K, Tanabe K, Ohnuki M, Narita M, Ichisaka T, Tomoda K, Yamanaka S (2007). Induction of pluripotent stem cells from adult human fibroblasts by defined factors. Cell.

[B40] Takahashi K, Yamanaka S (2006). Induction of pluripotent stem cells from mouse embryonic and adult fibroblast cultures by defined factors. Cell.

[B41] Wernig M, Meissner A, Foreman R, Brambrink T, Ku M, Hochedlinger K, Bernstein BE, Jaenisch R (2007). In vitro reprogramming of fibroblasts into a pluripotent ES-cell-like state. Nature.

[B42] Malynn BA, de Alboran IM, O'Hagan RC, Bronson R, Davidson L, DePinho RA, Alt FW (2000). N-myc can functionally replace c-myc in murine development, cellular growth, and differentiation. Genes Dev.

[B43] Wang X, McLachlan J, Zamore PD, Hall TM (2002). Modular recognition of RNA by a human pumilio-homology domain. Cell.

[B44] Glisovic T, Bachorik JL, Yong J, Dreyfuss G (2008). RNA-binding proteins and post-transcriptional gene regulation. FEBS Lett.

[B45] Chagnovich D, Cohn SL (1996). Binding of a 40-kDa protein to the N-myc 3'-untranslated region correlates with enhanced N-myc expression in human neuroblastoma. J Biol Chem.

[B46] Lazarova DL, Spengler BA, Biedler JL, Ross RA (1999). HuD, a neuronal-specific RNA-binding protein, is a putative regulator of N-myc pre-mRNA processing/stability in malignant human neuroblasts. Oncogene.

[B47] Crooks GE, Hon G, Chandonia JM, Brenner SE (2004). WebLogo: a sequence logo generator. Genome Res.

