# Supplementary material for: CompMoby: Comparative MobyDick for detection of cis-regulatory motifs
Source: BMC Bioinformatics. 2008 Oct 27;9:455. doi: 10.1186/1471-2105-9-455 (PMC2605473; doi:10.1186/1471-2105-9-455)
Supplement: Additional file 5 — Materials and methods for luciferase reporter assays. Materials and methods for ES cell culture and luciferase reporter assays in 3' UTR sequence analysis. [file 1471-2105-9-455-S5.pdf]

Materials and Methods for luciferase reporter assays:

Construction of Luciferase Constructs. Motifs were cloned into the NotI and XhoI sites in psiCheck™-2 vector (Promega, Madison, WI). Insert was generated by phosphorylating and annealing approximately 60nucleotide long oligonucleotides purchased from Integrated DNA technologies (Coralville, IA).

ES cell culture and luciferase reporter assays. V6.5 murine ES cells were cultured in DMEM high glucose media supplemented with 15% FBS, penicillin streptomycin, glutamine, non essential amino acids, and purified recombinant leukemia inhibitory factor (LIF) on 0.2% gelatin coated plates. ES cells were initially cultured on a feeder layer of irradiated MEFs and then weaned off MEFs by differential plating for approximately one hour. These MEF weaned ES cells were expanded 2-4 passages in 50% MEF conditioned media (media exposed to MEFs for approximately 1-2 days) and 50% fresh ES cell media. Media was changed daily.

On day 3, 4000 ES cells were plated in ES cell media in the same 96 well plate pretreated with 0.2% gelatin. Approximately 7 hours later, luciferase constructs were transfected into ES cells at a concentration of 200ng per well using FUGENE® 6 (Roche, Basel Switzerland) transfection reagent following the manufacturers protocol. The following day, 14-18 hours later, cells were lysed and luciferase assays were performed using a Dual-Luciferase® Reporter Assay System (Promega, Madison, WI) on a single automatic injection Mithras (Berthold technologies, Bad Wildbad, German) luminometer following the manufacturer's protocol. Transfection of each construct was performed in triplicate in each assay and a total of three assays were performed on three separate days. Empty vector was transfected in each plate in triplicate in both RA and ES conditions to be used for normalization purposes. Luciferase readings were taken as singlets. Ratios of Renilla luciferase readings to Firefly luciferase readings were taken for each experiment and triplicates were averaged. The average values of the tested constructs were normalized to the activity of the empty construct. Bars represent the averages of the normalized values with error bars indicating the range.
